# The Redox System in *C. elegans*, a Phylogenetic Approach

**DOI:** 10.1155/2012/546915

**Published:** 2012-07-31

**Authors:** Andrew D. Johnston, Paul R. Ebert

**Affiliations:** School of Biological Sciences, The University of Queensland, St Lucia, QLD 4072, Australia

## Abstract

Oxidative stress is a toxic state caused by an imbalance between the production and elimination of reactive oxygen species (ROS). ROS cause oxidative damage to cellular components such as proteins, lipids, and nucleic acids. While the role of ROS in cellular damage is frequently all that is noted, ROS are also important in redox signalling. The “Redox Hypothesis" has been proposed to emphasize a dual role of ROS. This hypothesis suggests that the primary effect of changes to the redox state is modified cellular signalling rather than simply oxidative damage. In extreme cases, alteration of redox signalling can contribute to the toxicity of ROS, as well as to ageing and age-related diseases. The nematode species *Caenorhabditis elegans* provides an excellent model for the study of oxidative stress and redox signalling in animals. We use protein sequences from central redox systems in *Homo sapiens*, *Drosophila melanogaster*, and *Saccharomyces cerevisiae* to query Genbank for homologous proteins in *C. elegans*. We then use maximum likelihood phylogenetic analysis to compare protein families between *C. elegans* and the other organisms to facilitate future research into the genetics of redox biology.

## 1. Introduction

Molecular oxygen is necessary for the survival of most complex multicellular organisms. The necessity of oxygen comes from its role in aerobic respiration, a process of extracting energy from food that is approximately 19 times more efficient than its anaerobic counterpart. In eukaryotes, aerobic respiration is carried out in the mitochondria (descendant of an aerobically respiring bacterium) by a series of electron transfer reactions that are coupled to the generation of a proton gradient. This proton gradient is used to generate the cellular fuel adenosine triphosphate (ATP). The residual energy of the spent electrons is consumed in the reduction of molecular oxygen (O_2_) to water (H_2_O). Aerobic respiration cannot occur without this last step, but the reliance on oxygen as the final electron acceptor poses a continual threat of oxidative damage to aerobically respiring organisms. 

**Figure 1:**
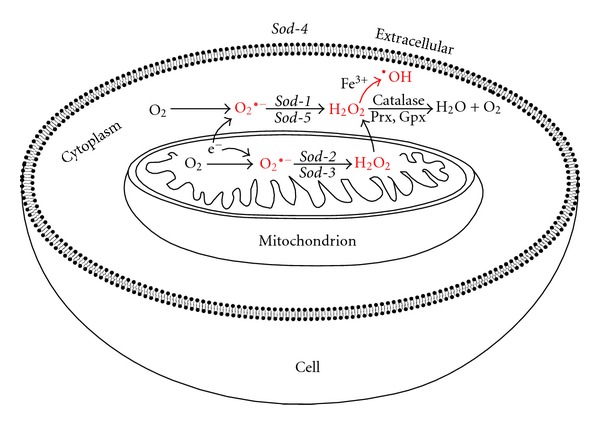
Simplified pathways of ROS production and antioxidant detoxification in a *C. elegans *cell. ROS molecules superoxide (O_2_^•−^), hydrogen peroxide (H_2_O_2_), and hydroxyl radical (^•^OH) are depicted in red. Antioxidants convert ROS to less reactive molecules. The five different superoxide dismutase enzymes convert superoxide to H_2_O_2_ and are depicted in their respective cellular compartments. Catalase, peroxiredoxin (Prx), and glutathione peroxidase (Gpx) convert H_2_O_2_ to H_2_O and O_2_. Rapid detoxification of H_2_O_2_ is necessary as it can become oxidised to form the potent free-radical ^•^OH. ROS can be damaging to DNA, RNA, proteins, and lipids, and high ROS levels can cause oxidative stress. Some antioxidant genes, such as catalase and *sod-3, *can be upregulated in response to oxidative stress.

The threat posed by oxygen comes largely from its conversion to the free radical superoxide (O_2_^•−^) rather than water [1]. Superoxide is a highly reactive short-lived ROS. Detoxification of superoxide and other ROS is performed by antioxidants, which convert ROS to less reactive molecules. The antioxidant enzyme superoxide dismutase (SOD) converts superoxide to water and hydrogen peroxide (H_2_O_2_), which is another ROS and a potent oxidising agent (see Figure 1) [2]. Under normal conditions, antioxidants help to prevent oxidative damage by using electrons to reduce ROS, thus inhibiting ROS from oxidising other molecules. However, an imbalance between ROS production and detoxification can result in oxidative stress. Numerous studies have found that high ROS levels are damaging to DNA, RNA, proteins, and lipids [3–6]. Additionally, oxidative (ROS) damage is thought to be one of the major causes of ageing, according to the free-radical theory of ageing [7]. However, the free-radical theory seems to conflict with recent findings regarding the role of ROS in redox signalling, findings that have unveiled an additional mechanism for oxidative toxicity besides simply macromolecular damage.

ROS are now known to do more than indiscriminately damage macromolecules; they function as important signalling molecules (reviewed by D'Autréaux and Toledano [8]). For instance, superoxide and H_2_O_2_ are part of a second messenger system involved in controlling subcellular redox states; modulating protein activation and turnover; regulating gene expression; and mediating extracellular signalling [9]. For this crucial messaging system to function, the levels of superoxide/H_2_O_2_ must be maintained at concentrations far below the level of toxicity. Therefore, ROS are unlikely to cause macromolecular damage under normal *in vivo* conditions. Consistent with this idea, recent studies have found that mitochondria produce superoxide/H_2_O_2_ at levels much lower than those previously estimated [10–12].

Jones [13] recently proposed the “redox hypothesis” as an alternative to the free-radical theory of ageing that accommodates recent discoveries in redox signalling. This hypothesis states that changes to redox state, rather than oxidative damage, cause aging and age-related diseases. The redox state of a cell, cellular compartment, or molecular system is a measure of the availability of chemically reactive electrons. In the reducing state, such electrons are more abundant whereas in the oxidising state they are less abundant. With regards to oxidative stress, the redox hypothesis suggests that an increase in ROS levels can be deleterious if the resulting oxidative shift in redox state causes a disruption to redox signalling.

To date much of what has been discovered in redox biology has resulted from work in *E. coli, S. cerevisiae*, mammals, and plants. While unicellular organisms such as *E. coli *and *S. cerevisiae *offer obvious advantages as model organisms due to their relative simplicity, short generation times, ease of culturing and maintenance, and so forth, they cannot be used to study systems/mechanisms unique to multicellularity in general, and animals in particular. Although not to the same extent, *C. elegans* offers similar advantages to working with the simple unicellular systems above, with the addition of being a multicellular, metazoan system allowing for much more of what is discovered in this organism to be extrapolated to research in other animals, including humans. This article seeks to scope out the redox proteins/systems of *C. elegans* as a resource for future work on redox signalling and oxidative stress within this model organism. In addition, this article will briefly explain the function of each protein family and how it relates to redox signalling and oxidative stress, with specific mention of what has been discovered in *C. elegans.*

## 2. Redox State, Redox Signalling, and Oxidative Stress

### 2.1. Redox-Sensitive Cysteine Switches

**Figure 2:**
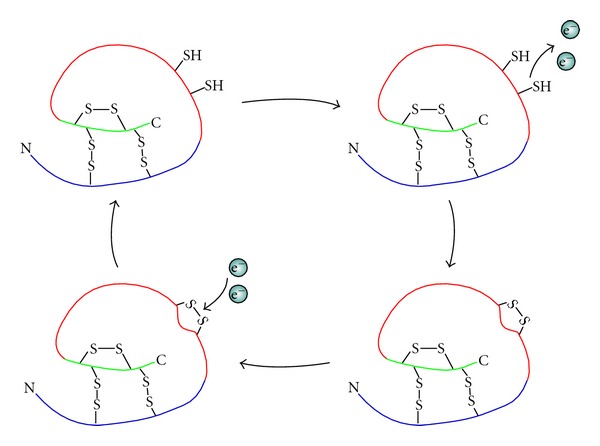
Cartoon of protein with redox-sensitive thiol switch. Structural disulfides can be seen on the inside of this protein, protected from redox reactions (those attached to the green and blue segments). A redox-sensitive thiol/disulfide switch is depicted on the protein surface (attached to the red segment). When reduced the redox-sensitive switch is in its dithiol state, however, when these thiols become oxidised (i.e., electrons are removed by an oxidant such as ROS) they bond to form a disulfide, altering the structure and/or function of the protein.

 The majority of available reactive electrons in a biological redox system are found in cysteines (as in the abundant tripeptide glutathione). Cysteine is an amino acid with a thiol (sulfur) group that is easily oxidized. When oxidised, two thiols in close proximity to one another can bond to form a disulfide. Formation of disulfides is important in protein folding and maintaining protein structure, however, a small fraction of thiols have another function: redox-sensitive switches (see Figure 2) [14–18]. These redox-sensitive thiol switches are generally found at the surface or in the active sites of proteins. Change from a thiol state to a disulfide can alter a protein's shape and function. The propensity for a redox-sensitive switch to be in one state or the other (thiol or disulfide) is dependent on the redox state of the cellular compartment and/or redox system to which it belongs. Therefore, the activity and conformation of a large number of proteins can be altered by changes in redox state [13].

### 2.2. The Redox State as a Signal

Redox signalling relies on oxidants and reductants that react preferentially with redox sensitive cysteines. Methionine also contains a redox active sulfur and is used in redox signalling [19], but this occurs to a lesser extent and is not discussed in this article. The most important oxidants that participate in signalling-related modification of cysteine residues are hydrogen peroxide (H_2_O_2_), a reactive oxygen species, and nitric oxide (NO), a reactive nitrogen species [20]. The focus of this article will be H_2_O_2_ as the signalling role of NO has been reviewed extensively [21]. Although a potent oxidant, the signalling role of H_2_O_2_ is primarily limited to redox-sensitive cysteine and methionine residues [22–24]. The fact that much of the cellular H_2_O_2_ is formed via the dismutation of superoxide (O_2_^•−^) by SOD enzymes means that the amount of superoxide produced directly contributes to the levels of H_2_O_2_ in a cell or cellular compartment [9]. This gives superoxide an important indirect role in redox signalling. In animals, superoxide is primarily generated by NADPH oxidase (NOX) [25], Coenzyme Q_10_ [9, 26], and Complex I and III [12] of the mitochondrial electron transport chain. 

**Figure 3:**
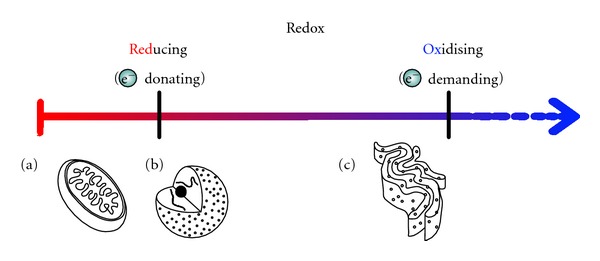
Redox state of between different cellular compartments. Redox state is a measure of the amount of electrons available for redox reactions. In the reducing state electrons are donated, whereas in the oxidising state electrons are removed. The mitochondria and nucleus are reducing (a and b). The endoplasmic reticulum is more oxidising (c).

The difference in redox states between organelles results from differences in the ratio between H_2_O_2_ and other thiol oxidants and various disulfide reductants/antioxidants. Because the redox state can alter protein conformation and reactivity, it can be used to activate or inactivate protein function. For example, the redox state of two different cellular compartments regulates DNA binding of the nuclear factor erythroid 2-related factor 2 (Nrf-2) transcription factor [27]. Nrf-2 is activated in the cytoplasm by an oxidative signal that results in translocation to the nucleus. However, in order to bind to DNA in the nucleus, a redox-sensitive cysteine must be reduced. This demonstrates specificity in redox signalling between different cellular compartments, for which the redox state of the compartment must be appropriate to its role (see Figure 3) [28]. 

### 2.3. Major Redox Systems: Glutathione, Thioredoxin, and Cysteine

**Figure 4:**
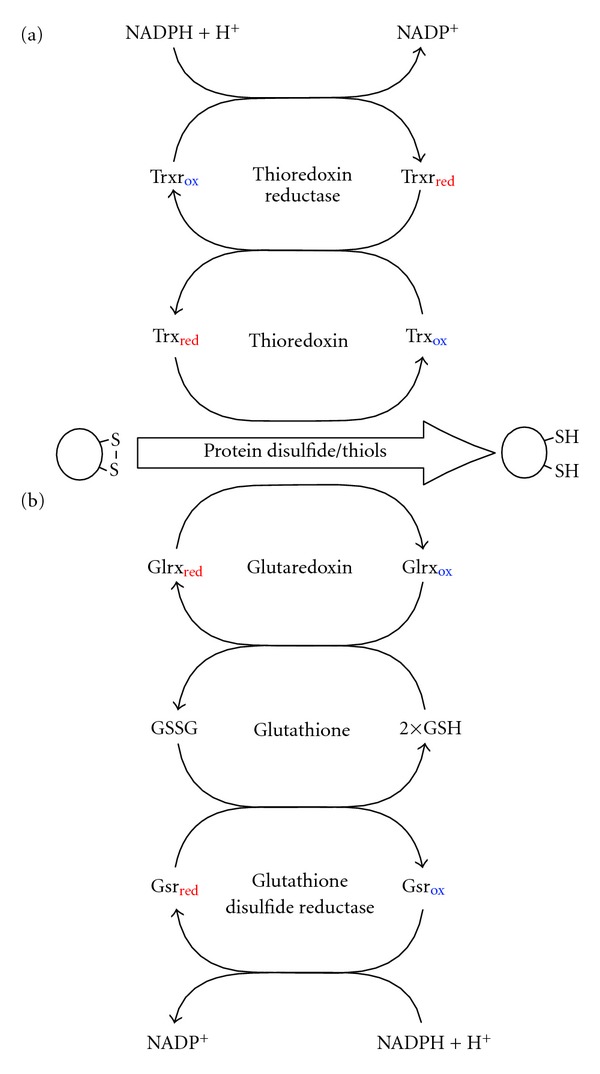
Glutathione-glutaredoxin and thioredoxin redox regulation systems. (a) Thioredoxin receives electrons from thioredoxin reductase and uses these to directly reduce protein disulfides. (b) Glutathione receives electrons from glutathione disulfide reductase; however, reduction of protein disulfides by glutathione is mediated through glutaredoxin. These two systems have similar functions, although there is evidence that indicates they each interact with different subsets of proteins. Arrows represent a change in redox state (loss or gain of electrons).

Two central thiol/disulfide couples work in the reduction of protein disulfides as counterparts to H_2_O_2_ and other oxidising agents to control redox state: the glutathione/glutathione disulfide (GSH/GSSG) couple (mediated through glutaredoxins) and the active site dithiol/disulfide of thioredoxins (Trx_red_/Trx_ox_; see Figure 4) [29]. Glutathione and thioredoxins each interact with a different subset of proteins thus forming distinct redox systems. The redox state of one of these systems may differ from the other even in the same cellular compartment [27, 30–34]. As well as glutathione and thioredoxin working as reducers of protein disulfides, a third thiol/disulfide couple, cysteine/cystine, has also been proposed by Jones et al. [32] as a possible oxidiser of protein dithiols used in redox regulation and signalling. Changes to the ratios of these three redox couples have been observed in various disease states, and it is possible that a gradual loss of redox state homeostasis over time contributes to ageing and age-related diseases [13]. 

### 2.4. Redox State versus Transient Local Redox Signalling

The redox state of the various redox systems in a cell or cellular compartment must normally reside within a narrow range, not only to maintain the constitutive signals resulting from the homeostatic redox state itself, but also to allow for meaningful thresholds, where a change in redox state outside the typical range of a cellular compartment can be used to signal a change in metabolism, environment, or stress. In addition to global signals at the level of an entire organelle, generation of H_2_O_2_ with no measureable effect on overall redox state of the various redox systems in a cell or cellular compartment may still have a very real effect on proteins in close proximity to the site of generation, resulting in a transient local signal—an idea explored more fully by Dwivedi and Kemp [35]. An example of modulation of signalling by transient local changes rather than a global shift in redox state is altered protein phosphorylation [36] resulting, for example, from the inactivation of protein tyrosine phosphatases [37, 38], MAP kinase phosphatases [39], and PTEN [40]. Within the redox hypothesis paradigm, much of the toxicity of oxidative stress could result from an oxidative shift in redox state within one or more cellular compartments. This shift would likely disrupt transient redox signalling as well as perturb the regular function of redox regulated proteins within these compartments. The end result could still be pathological oxidative damage to cellular components even though the cause could be indirect.

## 3. Phylogenetic Analysis

### 3.1. Thiol/Disulfide Redox Regulators

#### 3.1.1. Thioredoxin and Related Proteins

 Thioredoxin (TRX) was first discovered in *Escherichia coli* as a hydrogen donor for ribonucleotide reductase [41, 42]. Since the initial characterisation, TRX proteins have been recognized as more general disulfide reductases that are found in all phylogenetic domains of life. TRX proteins have a distinct structure that encompasses the active site dithiol known as the “thioredoxin fold.” This domain is also found in a variety of related proteins including glutaredoxin, protein disulfide isomerase, peroxiredoxin, and glutathione S-transferase [43]. Each of these protein families is discussed in other sections of this article. The characteristic CGPC active site dithiol motif can be oxidised to a disulfide to release electrons that are used to reduce redox-sensitive disulfides within a wide range of target proteins [44]. 

TRX proteins were long thought to be primarily involved in restoration of redox-sensitive disulfides to their reduced state after being oxidised by ROS. In particular, ROS scavengers such as peroxiredoxin require the activity of TRX proteins for their regeneration. However, the role of TRX as a disulfide reductase is now known to be important for immune signalling [45], regulating transcription factors [46], and modulating cellular signalling [47]. 

**Figure 5:**
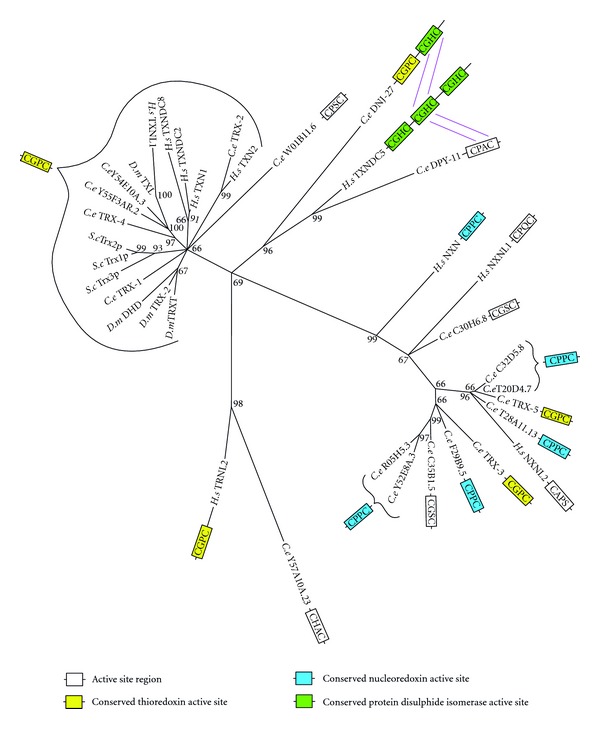
Maximum likelihood majority-rule bootstrap consensus tree of thioredoxin (TRX) and nucleoredoxin (NXN) proteins. A blast (blastp) search of known *Homo sapiens* (*H.s*), *Saccharomyces cerevisiae* (*S.c*), and *Drosophila melanogaster *(*D.m*) proteins was undertaken to identify all the homologs within these three species. The proteins from these species were then blasted against *Caenorhabditis elegans* (*C.e*) to identify homologues in the Genbank database. Identified sequences with significant expected value (≤~1E − 10) were used to generate a multiple sequence alignment (MSA) via ClustalW 2.1. The MSAs were then trimmed and used to produce a maximum likelihood majority-rule bootstrap consensus tree inferred from 1000 replicates via Mega 5.10. Proteins with sequences greater that ~350aa that were more closely related to other families discussed in this paper, such as GRX or PDI, were removed from this analysis. Some proteins fit equally well with TRX and TRX-related families, for example, DPY11 can be found in both the TRX and protein disulfide isomerase (PDI) trees.

Sequence comparisons and phylogenetic analysis revealed that *C. elegans* possesses twenty proteins closely related in sequence and length to TRX proteins in yeast, humans and fruit flies. Seven of these twenty proteins contain the characteristic CGPC active site sequence required for TRX activity. Five of these CGPC containing proteins, TRX-1, TRX-2, TRX-4, Y45E10.A, and Y55F3AR.2, are closely related to proteins of known TRX activity in *S. cerevisiae, H. sapiens, and D. melanogaster*. Human cytosolic thioredoxin 1 (TRXN1) has roles in the activation of transcription factors activator protein-1 (AP-1) [48] and nuclear factor kappa B (NF*κ*B) [49, 50], as well as in immune signalling [45, 51]. Although orthology is unclear between human TRXN1 and *C. elegans* TRX proteins (Figure 5), both human TRXN1 and *C. elegans* TRX-1 are cytoplasmic [52]. TRX-1 is expressed in intestinal cells as well as the ASJ pair of neurons and modulates adult lifespan extension induced by dietary restriction [53]. Human mitochondrial TRXN2, for which there is likely orthology with *C. elegans* TRX-2, is part of a mitochondria-dependent superoxide/TRXN2/apoptosis signal-regulating kinase 1 (ASK-1) apoptosis signalling pathway [54]. In *C. elegans* interactions of TRX-1 and TRX-2 with exonuclease 3 (EXO-3) and *C. elegans* p53-like protein (CEP-1), appear to play a role in neural structure and function as well as ageing [55]. Some of the functions of TRX are redox independent. For example, TRX-1 modulates the activity of the insulin-like neuropeptide DAF-28 in ASJ sensory neurons in the head during *C. elegans* dauer formation. This function was retained even after the redox activity of the protein was disrupted by replacing the two Cys residues of its active site with two Ser residues [56]. In regard to thioredoxin-like (TRXL) proteins, the close relationship found between Y45E10.A and Y55F3AR.2 and the thioredoxin-like proteins of humans (TXNL1) and *D. melanogaster* (TXL) suggests possible orthology. Functions of the TRXL proteins are yet to be determined. 

The other two *C. elegans* homologs containing the CGPC sequence, TRX-3 and TRX-5, were found as part a clade containing nucleoredoxin (NXN) and related proteins. Humans possess a single NXN, which contains a CPPC active site, and two nucleoredoxin-like proteins (NXNL1 and NXNL2). NXN (reviewed in [57]) has been shown to function as a redox regulator of gene expression [58] and a negative regulator of toll-like receptor signalling [59]. It also sustains Wnt/ß -Catenin signalling [60]. Six out of the nine *C. elegans* proteins within the NXN clade contain the CPPC NXN active site sequence, suggesting a possible expansion of the NXN subfamily. Proteins within this NXN clade are also closely related to the 16-Kilodalton class of thioredoxins described in the parasitic nematode *Brugia malayi* [61]. 

#### 3.1.2. Glutathione

Reduced glutathione (GSH) is a tripeptide consisting of glycine, cysteine and glutamic acid. GSH synthesis is performed in a two-step ATP-dependent process. In the rate-limiting first step *gamma*-glutamylcysteine synthetase (GCS; see Table 1) synthesises *gamma*-glutamylcysteine from L-glutamate and cysteine. In the second-step glutathione synthetase (GSS; see Table 2) adds glycine to the C-terminal of *gamma*-glutamylcysteine. These enzymes are highly conserved in eukaryotes (Tables 1 and 2) and even in prokaryotes (not shown). 

Glutathione plays an essential role in antioxidant defence as a source of electrons for antioxidant enzymes such as glutaredoxins and peroxidases [63]. The high (millimolar) concentrations of glutathione in the cell ensure an abundance of electrons for these antioxidant systems and thus provide a robust buffer against oxidative shifts in redox state [64]. GSH also serves as a reversible cysteine adduct. Glutathione S-transferases (GSTs) can form mixed disulfides between glutathione and redox-sensitive cysteine thiols of proteins. This activity can be used to regulate protein activity and under oxidizing conditions can prevent irreversible oxidation of thiols to sulfinic (SO_2_H) and sulfonic acid (SO_3_H) oxoforms [65, 66]. GST can also conjugate glutathione to xenobiotic compounds as part of a detoxification response [67] and to a fatty acid in the synthesis of prostaglandin hormone [68, 69].

 The ratio of reduced glutathione to glutathione disulfide within a cellular compartment, that is, [GSH]^2^/[GSSG], determines its redox state. High [GSH]^2^/[GSSG] ratios such as those found in the mitochondria, cytoplasm, and nucleus ensure that the majority of redox-sensitive protein switches within these compartments are in the reduced (–SH) state [77]. Maintenance of the proper [GSH]^2^/[GSSG] ratio ensures redox homeostasis, whereas changes to this ratio provide a simple means to adjust the redox state between compartments as well as within compartments under different physiological conditions. For example, changes in redox state have been found to trigger responses associated with defence against particular biotic or abiotic stressors [78]. In plants, changes to the cellular glutathione pool havebeen shown to elicit pathogen resistance responses [79, 80]. These examples demonstrate that global changes to protein activity and widespread changes to signalling can be achieved quite readily by simply changing the redox set point within a cellular compartment.

#### 3.1.3. Glutathione Disulfide Reductase and Thioredoxin Reductase

**Figure 6:**
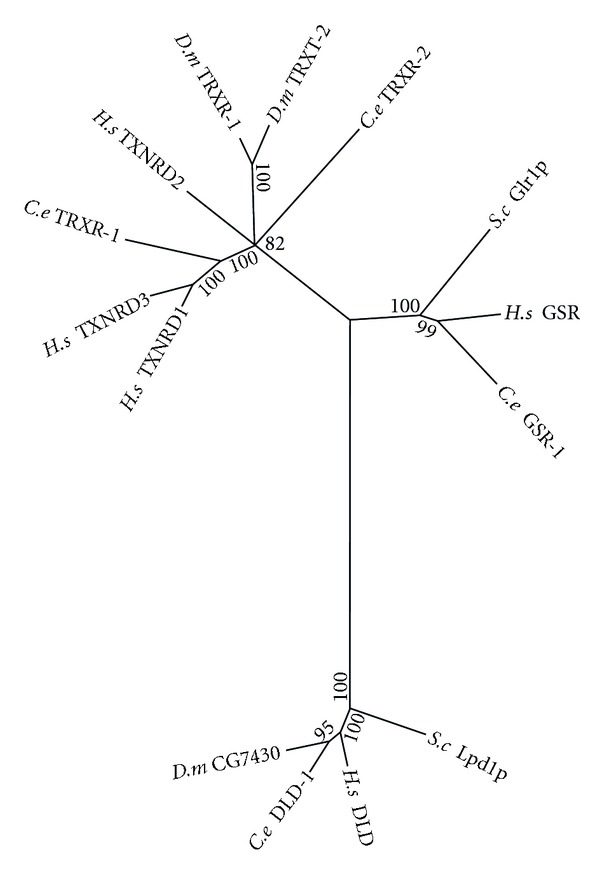
Maximum likelihood majority-rule bootstrap consensus tree of thioredoxin reductase (TRXR), glutathione disulfide reductase (GSR) and dihydrolipoamide dehydrogenase (DLD) proteins. *C. elegans *possess two TRXR: mitochondrial TRXR-1 and cytosolic TRXR-2. In addition *C. elegans *contain a single glutathione disulfide reductase (GSR) and the closely related DLD. See Figure 5 for details of how the sequences were identified and processed as well as how the phylogenetic analysis was carried out.

 When oxidised, the reduced (thiol) states of glutathione and TRX enzymes are restored by glutathione disuflide reductase (GSR) and TRX reductase (TRXR), respectively, using electrons obtained from NADPH (Figure 4) [81]. The maximum likelihood tree of this family forms three clades (Figure 6). GSR is absent from *D. melanogaster* and has been substituted by a novel glutaredoxin-thioredoxin reductase fusion protein (TRXR-1) [82]. *S. cerevisiae* possess proteins with TRXR enzymatic activity, however, these proteins are evolutionarily unrelated to the animal forms and are thus not represented on the phylogenetic tree [83]. Interestingly, TRXR-1 is the only selenocysteine containing protein in *C. elegans*, and both TRXR-1 and GSR-1 are essential for proper moulting [84].

The third clade of this protein family is composed of dihydrolipoamide dehydrogenase (DLD) orthologs. DLD is similar in structure to both TRXR and GSR, however, its functions are quite different. DLD is a component of various protein complexes located within the mitochondrial matrix, including pyruvate dehydrogenase, alpha-ketoglutarate dehydrogenase, and the branched chain amino acid-dehydrogenase complexes as well as the glycine cleavage system. In these complexes DLD is required for regeneration of oxidised lipoamide from the reduced dihydrolipoamide cofactor. The pyruvate dehydrogenase and branched chain amino acid-dehydrogenase complexes (2-Oxo acid dehydrogenase complexes) are thought to play a role in redox regulation via the reduction of thioredoxins [85]. In addition, the alpha-ketoglutarate dehydrogenase complex has been found to be a generator of H_2_O_2_ [86, 87].

#### 3.1.4. Glutaredoxin

**Figure 7:**
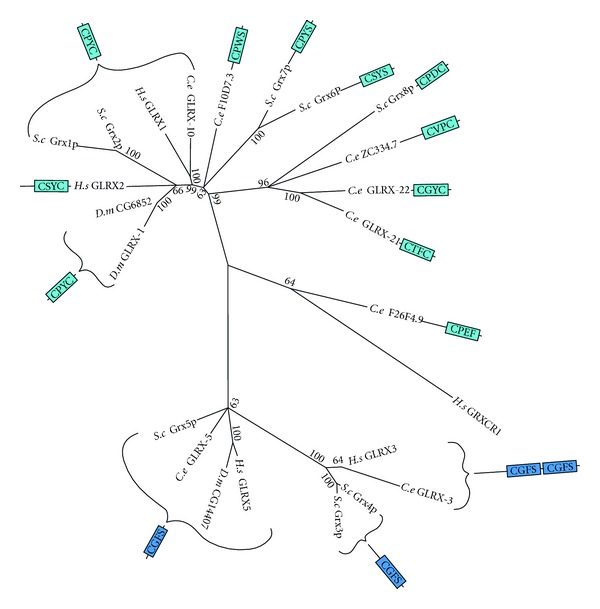
Maximum likelihood majority-rule bootstrap consensus tree of glutaredoxin (GLRX) proteins. Dark blue signifies the conserved active site of monothiol GLRX. Light blue signifies proteins of greater similarity to known dithiol GLRX, regardless of whether or not two cysteines are actually is present in the region aligned to the active sites. See Figure 5 for details of how the sequences were identified and processed as well as how the phylogenetic analysis was carried out.

Glutaredoxin (GLRX) uses electrons extracted from GSH to reduce redox-sensitive disulfides of a variety of proteins, thereby modulating enzyme activity [88]. GLRX can also carry out oxidative cysteine glutathionylation of proteins resulting in protein-glutathione mixed disulfides [78], and the reverse reaction, deglutathionylation (i.e., the reduction of the mixed disulfides), restoring the protein to its unmodified form. GLRX enzymes come in two forms: a monothiol form that contains a single cysteine in the active site and a dithiol form that contains two cysteines in the active site. These two forms differ in function and can be seen as distinct clades in the phylogenetic tree (Figure 7). The reduction of protein disulfides as well as the oxidative formation of protein-glutathione-mixed disulfides are both catalysed via dithiol mechanisms, whereas reductive deglutathionylation is performed by a monothiol mechanism [89].

One of the GLRX clades contains only monothiol proteins with the CGFS active site sequences, whereas the other clade contains mostly dithiols with a variety of active site sequences, as well as a few proteins with a single cysteine active site. The mammalian GLRX3 (PICOT), likely ortholog of *C. elegans* GLRX-3, has been characterised as an iron-sulfur binding protein possibly regulated by ROS and reactive nitrogen species [90]. GLRX3 is essential for embryonic development, postembryonic growth, and heart function [91]. Human GLRX1 has a number of roles including the regulation of redox signal transduction and protein translocation [92], caspase-3 signalling in tumor necrosis factor-*α*-induced cell death [93], and angiotensinII redox signalling via glutathionylation of Ras [94]. *C. elegans *GLRX-10 is closely related to human GLRX1, both of which are nested within a subclade of dithiol GLRX enzymes, all of which except GLRX2 contain the CPYC active site sequence. F10D7.3 is somewhat similar to Grx6p and Grx7p from *S. cerevisiae*, although the difference in size and active site makes orthology unlikely. GLRX-21, GLRX-22, and ZC334.7 were found to group closely with *S. cerevisiae* Grx8p, but again differences in size (particularly the larger size of ZC334.7) and the sequence of their dithiol active sites makes orthology unlikely. To date very little work on GLRX proteins has been performed in *C. elegans*. Worth mentioning, however, is a paper published in 2010 which found that GLRX-21 functions in the prevention of selenium-induced oxidative stress [95].

#### 3.1.5. Protein Disulfide Isomerase

The protein disulfide isomerase (PDI) protein family is composed of a large and diverse group of enzymes, most of which contain at least one TRX-like domain with a CxxC active site motif. PDI enzymes reside in the endoplasmic reticulum (ER) where their usual function is to catalyse protein folding. The active site cysteines of PDI are used in thiol-disulfide exchange between cysteine residues of the substrate proteins. This PDI thiol-disulfide exchange enables proteins to rapidly aquire the correct configuration of structural disulfide bonds required to achieve their native structure [96]. PDI functions in four different chemical reactions: (1) the oxidation of protein disulfides, using GSSG as the electron acceptor; (2) the reduction of protein disulfides, using GSH or NADPH as the electron donor; (3) the deglutathionylation of mixed disulfides; (4) the isomerization (rearrangement) of intra-molecular disulfides. These functions of PDI proteins require the more oxidised redox state of the ER [70, 97]. In addition to passively relying on a more oxidised redox state, it has also been suggested that some PDI proteins may play a role in redox regulation [62].

In *C. elegans*, Karala et al. [98] analysed and compared the activities of PDI-1, PDI-2, and PDI-3 and found that all three displayed thiol-disulfide exchange activity, but that each showed a difference in reactivity towards various protein substrates. RNAi knockdown of the *pdi-2* and *pdi-3* genes results in an unflolded protein response, which suggests PDI-2 and PDI-3 are indeed required for proper protein folding [83]. Additionally, Winter et al. [99] studied PDI-1, PDI-2, and PDI-3 and found that PDI activity is required for embryonic development and proper formation of the extracellular matrix. 

**Figure 8:**
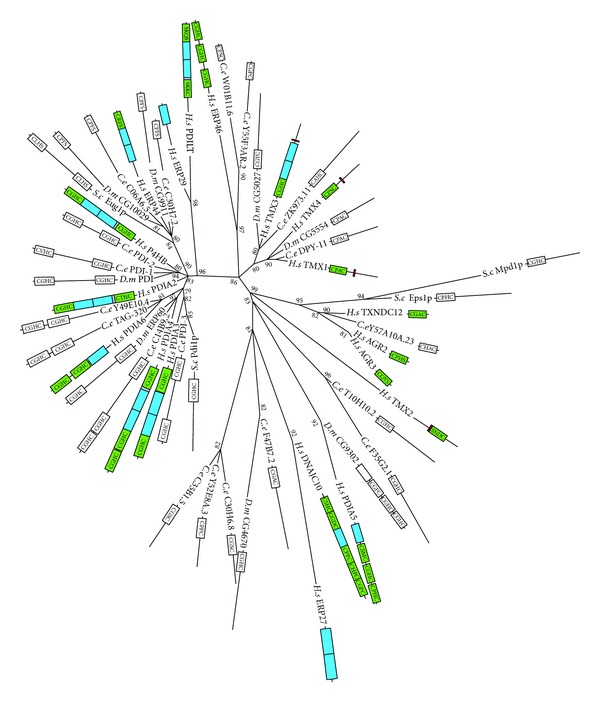
Maximum likelihood majority-rule bootstrap consensus tree of protein disulfide isomerase (PDI) proteins. Green boxes are likely TRX-like fold in human PDI; blue are likely noncatalytic thirodoxin domains (according to Hatahet and Ruddock [62]). White boxes show CxxC catalytic motifs, in *C. elegans* and *D. melanogaster*. Although domain structure cannot be inferred from sequence alone, it is likely that proteins that share similar sequences, catalytic motifs, and length also share a similar domain structure and in some cases are likely orthologous. See Figure 5 for details of how the sequences were identified and processed as well as how the phylogenetic analysis was carried out.

Comparision of PDI sequences and phylogentic analysis revealed a number of unnamed proteins with likely othology to known human PDIs, as well as a few small gene expansion events both in *C. elegans* and human. The end result is 19 proteins in both *C. elegans* and human, but only 8 in *D. melanogaster* and 4 in *S. cerevisiae *(see Figure 8). Human P4HB (PDI/PDIA1), PDIA3 (ERp57), and PDIA4 (ERp72)—the probable orthologs of *C. elegans* PDI-2 (or PDI-1), PDI-3, and C14B9.2, respectively—all react readily with peptide dithiols *in vitro* to form disulfides [100]. *C. elegans* PDI-1 is peculiar in that the N-terminal active sites of all of its closely related homologs contain the characteristic PDI sequence CGHC, whereas in *C. elegans* the glycine has been replaced by a valine (CVHC). The similarity of *C. elegans* PDI-1 to human P4HB in size and sequence would suggest an orthologs relationship between these two proteins. However, human P4HB can be found as the beta subunit of prolyl 4-hydroxylase (P4H),a complex which hydroxylatesprolinetohydroxyproline in the production ofcollagen [101]. In *C. elegans*, the beta subunit of the P4H complex is PDI-2 [102, 103], making PDI-2 the more likely ortholog of P4HB, despite PDI-2 being ~100 amino acids shorter than *C. elegans* PDI-1 and human P4HB. Regarding the other PDI homologs, Ko and Chow [104] found that the DPY-11 protein of *C. elegans* that is a possible ortholog of human TMX1, is necessary for body and sensory organ morphogenesis, which they argue is due to its role in substrate modification in the hypodermis. In terms of redox signalling, human P4HB has been found to work antagonistically with TRXN1 in the regulation of nuclear factor kappa-light-chain-enhancer of activated B cells (NF-*κ*B)-dependent gene expression: TRXN1 actives the NF-*κ*B pathway, whereas P4HB expression suppresses NF-*κ*B activity in a dose-dependent manner [105]. Other possible PDI orthologous relationships involving *C. elegans* include human TMX3, *D. melanogaster* CG5027, and *C. elegans* ZK973.11; human TXNDC12 (ERp18) and *C. elegans* Y57A101.23; *D. melanogaster* CG4670 and *C. elegans* F47B7.2; human PDIA6 (p5), *C. elegans* TAG-320 and *C. elegans* Y49E10.4 (see Table 3). Although, whether any of these proteins participate in redox signalling remains to be investigated.

### 3.2. Superoxide/H_2_O_2_ Generation and Removal

#### 3.2.1. NADPH Oxidase

The NADPH oxidase (NOX) system was first described as a system used by mammalian phagocytes in the production of superoxide as a response to infection by microorganisms [106]. The core enzyme of this microbial defence system is NOX2, which—under the regulation of its p22phox, p47phox, p40phox, p67phox, and RAC subunits—catalyses the formation of large amounts of superoxide, which in turn is converted to additional reactive oxygen species. The resulting high ROS levels results in the death of invading microbial pathogens. A total of seven NOX homologues exist in mammals, including NOX1 through 5 and the dual oxidases DUOX-1 and DUOX-2. Most of these homologs generate much lower of levels of ROS than NOX2 and are found in a much wider range of cell types. Lambeth [25] presents a case for the importance of NOX proteins in the generation of ROS signals, but this hypothesis has not yet been rigorously tested. 

**Figure 9:**
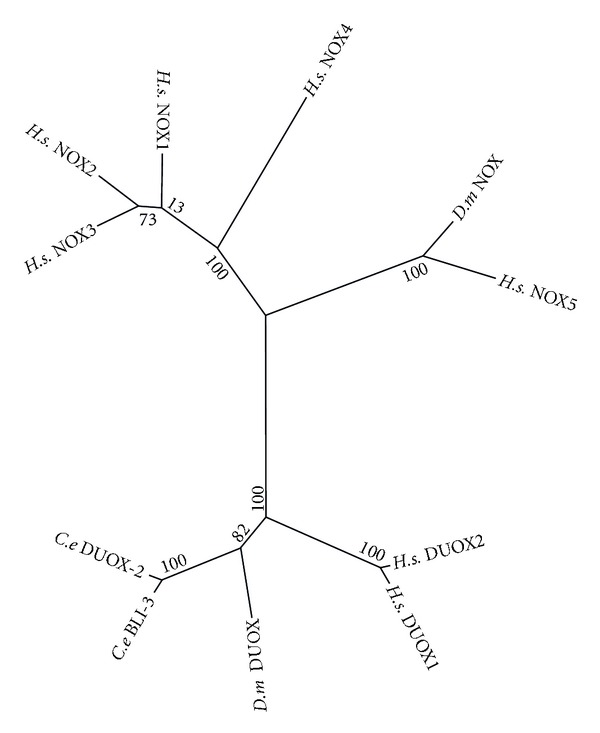
Maximum likelihood majority-rule bootstrap consensus tree of NADPH oxidase (NOX)/dual oxidase (DUOX) proteins. See Figure 5 for details of how the sequences were identified and processed as well as how the phylogenetic analysis was carried out.

Most important to a discussion of NOX activity in *C. elegans* is the function of DUOX, as the only two NOX homologs in *C. elegans*, DUOX-2 and BLI-3, are related to the DUOX proteins of humans and *D. melanogaster* (Figure 9). These proteins contain an additional peroxidase domain not found in the NOX1 through 5. DUOX serves a dual role in both the generation of superoxide and catalysis of reactions in the extracellular matrix using H_2_O_2_. The subunits used to regulate NOX2 are not used in the regulation of DUOX enzymes. Additionally, blastp searches do not reveal homology to any of the NOX2 regulating subunits in *C. elegans*. It is important to note the DUOX has not been implicated in ROS signalling, and there is evidence to suggest that in mammals they instead play a role in the biosynthesis of thyroid hormones in the extracellular matrix [107]. In *C. elegans*, the DUOX homolog BLI-3 functions in tyrosine cross-linking in the extracellular matrix [108, 109]. Further research may yet reveal additional mechanisms for the DUOX homologs in *C. elegans*.

#### 3.2.2. Superoxide Dismutase

 SOD proteins are generally regarded as antioxidants responsible for eliminating the ROS superoxide. An alternative view is that these enzymes generate H_2_O_2_ for use in redox signalling [110]. In this regard, the levels of SOD activity could be important in regulating H_2_O_2_ levels. A signalling role for SOD goes well beyond the popular view that SOD is responsible for the complete removal of superoxide from cellular compartments for the sole purpose of preventing oxidative damage. 

Much of the recent research on SOD enzymes has focused on their possible role in the ageing process, in experiments designed to test the free-radical theory of ageing. In some cases, decreasing levels of SOD have been shown to shorten the lifespan of yeast [111–114], fruit flies [115], and mice [116], but this is not uniformly the case. In fact, an analysis of the entire *sod* gene family in *C. elegans* revealed that both increasing and decreasing expression of the *sod* genes had little effect on lifespan [117]. When *sod* gene expression is experimentally increased, lifespan is either unaltered or decreased [117–120]. A report from Y. Honda and S. Honda [121] showed that increased expression of *sod-1* and *sod-2* extended the lifespan of *C. elegans*, but that this was not due to decreased oxidative damage. While results are inconsistent between species and are not even consistent between experiments on a single species, it is clear that the view of SOD as an eliminator of ROS that would otherwise limit lifespan is much too simplistic.

**Figure 10:**
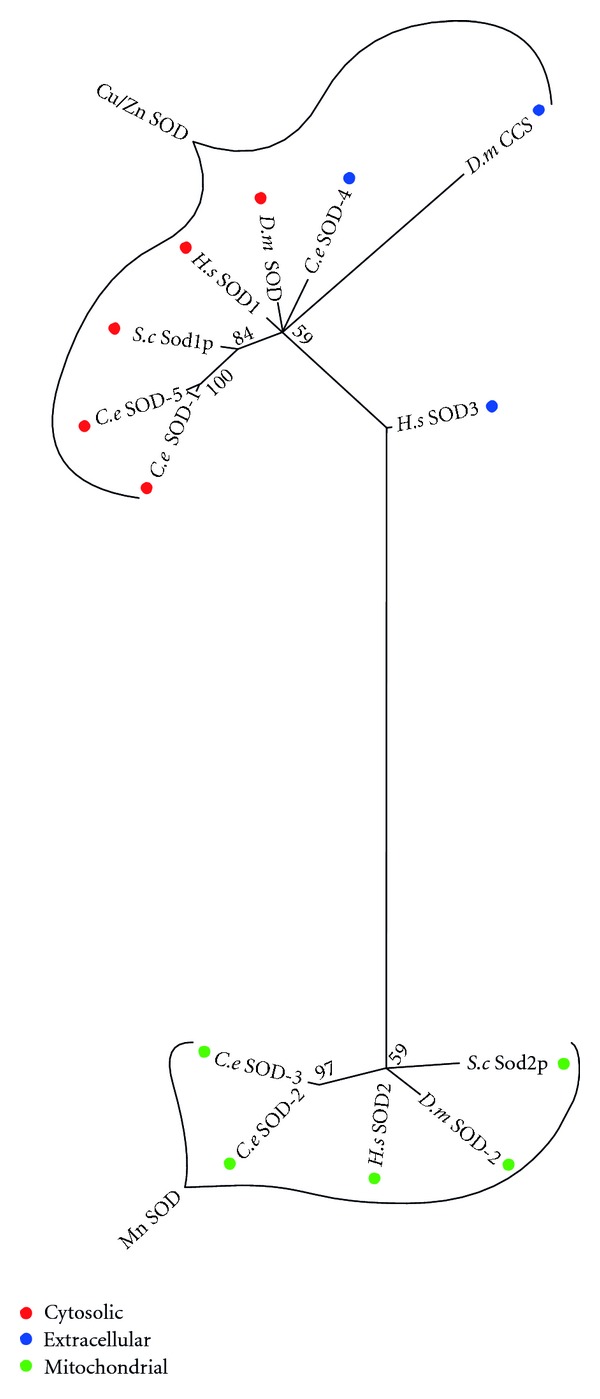
Maximum likelihood majority-rule bootstrap consensus tree of superoxide dismutase (SOD) proteins. See Figure 5 for details of how the sequences were identified and processed as well as how the phylogenetic analysis was carried out.

Two distinct classes of SOD enzymes exist within eukaryotes: copper/zincSOD (Cu/Zn SOD), found in the cytosol or extracellular matrix [122]; manganese SOD (Mn SOD), found in the mitochondria [123]. Phylogenetic analysis clearly shows two main clades corresponding to Mn and Cu/Zn enzymes (Figure 10). Unlike the other three species in the analysis, each of which has only a single Mn SOD, there has been a duplication of the gene in *C. elegans*, *sod-2* and *sod-3.* The same is true of the human Cu/Zn *sod-1*, in that human and the other two species have a single gene that corresponds to a pair of genes in *C. elegans*, *sod-1* and *sod-5*. The last *sod* gene in *C. elegans*, *sod-4*, corresponds to a single gene in each of human and *D. melanogaster*. It is an extracellular Cu/Zn SOD [124], with a possible function is *daf-2* signalling [117].

#### 3.2.3. Glutathione Peroxidase

**Figure 11:**
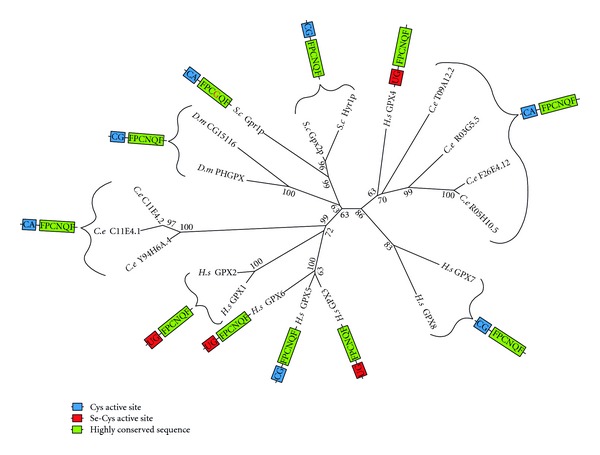
Maximum likelihood majority-rule bootstrap consensus tree of GPX proteins. See Figure 6 for details on the phylogenetic analysis.

The glutathione peroxidases (GPX) were first characterised as a family of proteins that reduce H_2_O_2_ to H_2_O using GSH as the electron donor [125]. Humans and *C. elegans* both contain a large number of GPX proteins (8 and 7, resp.) compared to yeast (3 proteins) and *D. melanogaster* (only 2 proteins). Most of these appear to have arisen independently by gene duplications within the two taxonomic lineages (Figure 11). While five of the eight humans GPX contain a selenocysteine in their active site, not a single selenocysteine is found in any of the *C. elegans* GPX homologs.

Despite the relatedness of *C. elegans* proteins with proteins of known GPX activity, Vanfleteren [126] was unable to detect any GPX activity in *C. elegans* tissue. However, the *in vitro* assay used in this article only included GSH as a reducing substrate. Use of GSH appears to be limited to GPX enzymes that contain selenocysteine; the cysteine-containing GPX homologs of *C. elegans* likely use a peroxiredoxin-like mechanism with thioredoxin as their reducing substrate [127]. 

Numerous studies have implicated GPX proteins in redox signalling. For example, Faltin et al. [128] found that ROS signalling used in the early stage shoot organogenesis of plants is regulated by the GPX homolog PHGPx. In mammals, ROS regulation of lipopolysaccharide (LPS) signalling is modulated by GPX1 [129], while GPX1 deficiency is found to enhance proinflammatory cytokine-induced redox signalling [130]. Conversely, high levels of catalase and GPX activity have been found to diminish H_2_O_2_ signalling in human alveolar macrophages [131]. The *C. elegans* protein F26E4.12, a homolog of human GPX4 and plant PHGPx, regulates the peptide transporter PEPT-1 [132]. 

#### 3.2.4. Peroxiredoxin

Peroxiredoxins (PRDX) are found as homodimers in which the active site cysteines align in close proximity and form intermolecular dithiols/disulfides. PRDX disulfides function in the reduction of H_2_O_2_ to H_2_O for both antioxidant defence and mediation of ROS signalling [133, 134]. Active site disulfides formed in the reduction of H_2_O_2_ are reduced back to dithiols by the thioredoxin redox system. PRDX comes in three forms: “Typical” 2-Cys, “Atypical” 2-Cys, and 1-Cys. 

**Figure 12:**
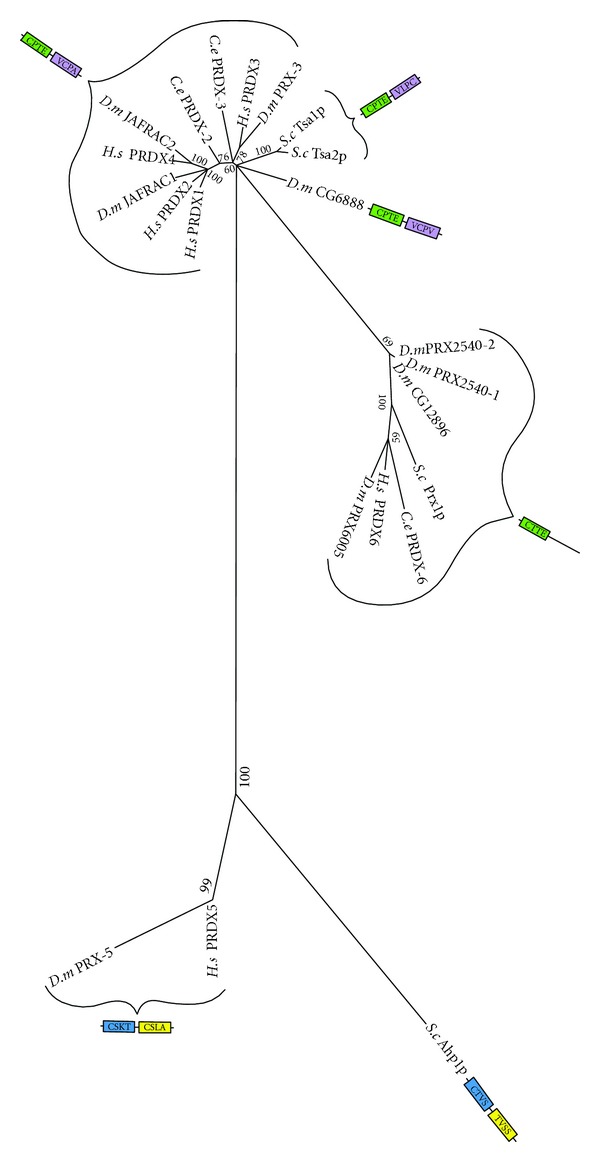
Maximum likelihood majority-rule bootstrap consensus tree of peroxiredoxin (PRDX) proteins.“Typical” 2-Cys PRDX is shown in green and purple; “Atypical” 2-Cys in blue and yellow; and 1-Cys in green. Active site cysteine regions highlighted in the same colour are those that aligned in the MSA. See Figure 5 for details of how the sequences were identified and processed as well as how the phylogenetic analysis was carried out.

Phylogenetic analysis showed that *C. elegans* have two typical 2-Cys PRDX, 1-Cys PRDX, but does not possess an atypical 2-Cys PRDX homolog (Figure 12). Human PRDX1 and PRDX2, two of the typical 2-Cys PRDX homologs to *C. elegans* PRDX-2, might participate in both intra- and extracellular signalling cascades by regulating levels of H_2_O_2_ [23]. The human mitochondrial typical 2-Cys PRDX3, which is closely related to *C. elegans* PRDX-3, participates in the regulation of apoptotic signalling. Little research has been done on the PRDX proteins of *C. elegans*. What is known is that PRDX-2 is necessary for normal growth and egg production in *C. elegans*, which Isermann et al. [135] argue is likely due to its role in peroxide signalling. Interestingly, loss of PRDX-2 actually increases resistance to some oxidative stress causing agents but results in a decrease in lifespan [136].

#### 3.2.5. Catalase

**Figure 13:**
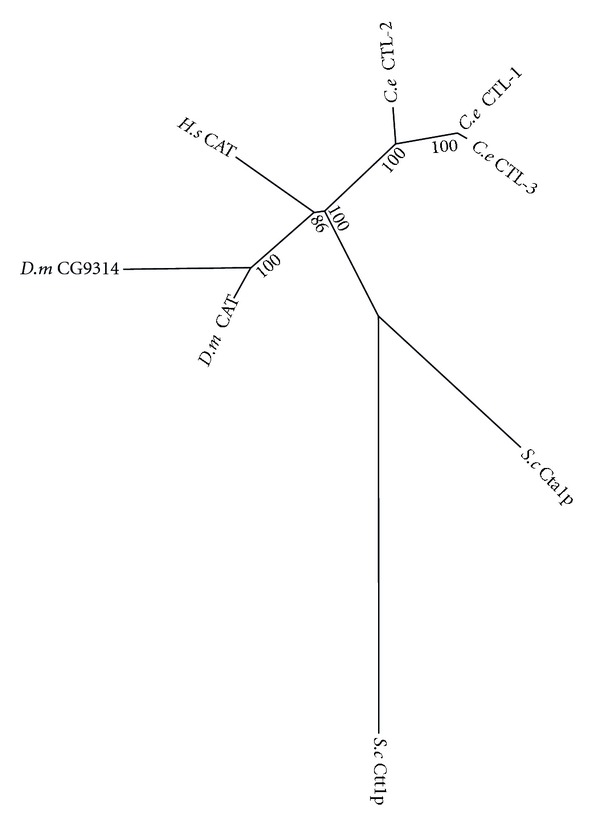
Maximum likelihood majority-rule bootstrap consensus tree of catalase proteins. See Figure 6 for details on the phylogenetic analysis.

Catalase functions in the decomposition of H_2_O_2_ to H_2_O and O_2_. Phylogenetic analysis shows a lineage specific expansion from one catalase to three in *C. elegans *(Figure 13). *C. elegans* CTL-1 is required for the extended adult lifespans of *daf-2*, *age-1*, and *clk-1* mutants [137]. CTL-2 is found in the peroxisomes of *C. elegans* [138] and a lack of this protein has been found to cause a progeric phenotype and adversely affect development/egg laying [139]. The *ctl-1* and *ctl-2* genes are both negatively regulated by DAF-2-mediated insulin signalling [140]. As such, DAF-2 signalling might result in an increase in the levels of H_2_O_2_, and thus a more oxidizing state.

## 4. Conclusion

Reactive oxygen species are thought to play a role in many diseases of ageing, including Parkinson's disease, Alzheimer's disease, heart failure, and myocardial infarction. This makes understanding their dual roles as oxidative stressors and signalling molecules highly significant. Important to this understanding is a clear description of the protein families that contribute to the generation and metabolism of ROS. Most of the known protein families that participate in redox biology are discussed in this article, but it is likely that additional, undescribed families of redox proteins remain to be discovered. It is striking that of the protein families that have been compared in this study, most show clear relationships between sequences, with no extreme examples of species-specific family expansion. *S. cerevisiae* frequently, and *D. melanogaster* sometimes, had smaller gene families than the other two species. 

 In addition to oxidative damage, higher ROS levels disrupt the regular function of redox regulators and their downstream effectors. It is, therefore, likely that at least some, if not many, of the toxic effects associated with oxidative stress are the result of disruption to redox signalling. Continued research into the various functions of the *C. elegans* redox proteins discussed in this article will help to achieve a better understanding of redox signalling, oxidative stress, and the relationship between these two biological phenomena. *C. elegans* and *H. sapiens* exhibited fairly conserved gene family structure, indicating that *C. elegans* will provide a medically relevant model of redox signalling.

## Figures and Tables

**Table 1:** Similarity between *C.e gamma*-glutamylcysteine synthetase and orthologues in *H.s., S.c.,* and *D.m*.

Species name	Protein name	Function	% identity	*E* value
*H.s.*	GCLC	*gamma*-glutamylcysteine synthetase	350/643 (54%)	0.0
*D.m.*	GCLC	*gamma*-glutamylcysteine synthetase	281/498 (56%)	0.0
*S.c.*	GSH1	*gamma*-glutamylcysteine synthetase	254/672 (38%)	2*e* − 138

*C.e*: *C. elegans*, *H.s*: *H. sapiens*, *S.c*: *S. cerevisiae*, *D.m*: *D. melanogaster.*

**Table 2:** Similarity between *C.e* glutathione synthetase and orthologues in *H.s., S.c*., and *D.m*.

Species name	Protein name	Function	% identity	*E* value
*H.s.*	GSS	glutathione synthetase	185/470 (39%)	3*e* − 95
*D.m.*	CG32495	glutathione synthetase	180/491 (37%)	8*e* − 102
*S.c.*	GSH2	glutathione synthetase	147/494 (30%)	1*e* − 56

*C.e*: *C. elegans*, *H.s*: *H. sapiens*, *S.c*: *S. cerevisiae*, *D.m*: *D. melanogaster*.

**Table 3:** Characterised protein disulfide isomerase and their *C. elegans* orthologs.

PDI homolog	Known functions	*C. elegans* ortholog (paralogs)
AGR2	Unknown	None

AGR3	Unknown	None

*ERp27*	*Unknown*	*(C30H6.8, C35B1.5, *
*DNAjc10*	*ER-associated protein degradation * *[70]*	* F47B7.2, Y52E8A.3)*

ERp29	Specialist ‘‘escort” chaperone [71] Thyroglobulin processing [72]	None

ERp46	Some P4HB functional redundancy Protective role against hypoxia [70]	(W01B11.6, Y55F3AR.2)

ERp44	Possible role in late-stage oligomerization reactions Possible role in late-stage, thiol-dependent, protein quality-control system or trafficking [70]	C06A6.5, C30H7.2

P4HB	Thiol-disulfide exchange catalysis Role in protein folding Beta subunit of P4H complex [70]	PDI-2, PDI-1

PDIA2	Expressed in pancreas and brain [73, 74] Thiol-disulfide exchange catalysis similar to P4HB Specific function still unclear	None

PDIA3	Involved in MHC class I folding as part of MHC I peptide-loading complex [75] Oxidative folding of glycoproteins	PDI-3

PDIA4	Thiol-disulfide exchange catalysis similar to P4HB Some PDIA3 redundancy Can form part of a complex with P4HB, PDIA6, ERdj3, BiP, CypB, HSP40, GRP94, GRP170, UDP glucosyltransferase, and SDF2-L1 [76]	C14B9.2

PDIA5	Unknown	(F35G2.1, T10H10.2)

PDIA6	Likely catalyze thiol-disulfide exchange similar to P4HB Likely role in protein folding	TAG-320, Y49E10.4

PDILT	Unknown	None

TMX1	ER transmembrane PDI Unknown function	DPY-11

TMX2	ER transmembrane PDI Unknown function	None

TMX3	ER transmembrane PDI Protein dithiol-disulfide oxidant	ZK973.11

TMX4	ER transmembrane PDI Unknown function	None

TXNDC12	Unknown	Y57A10A.23

Italic rowsare human proteins that share the same *C. elegans* paralogs.

## References

[1] Miwa S, St-Pierre J, Partridge L, Brand MD (2003). Superoxide and hydrogen peroxide production by *Drosophila mitochondria*. *Free Radical Biology and Medicine*.

[2] McCord JM, Fridovich I (1969). Superoxide dismutase. *The Journal of Biological Chemistry*.

[3] Mead JF (1976). Free radical mechanisms of lipid damage and consequences for cellular membranes. *Free Radicals in Biology*.

[4] Brot N, Weissbach L, Werth J, Weissbach H (1981). Enzymatic reduction of protein-bound methionine sulfoxide. *Proceedings of the National Academy of Sciences of the United States of America*.

[5] Demple B, Linn S (1982). 5,6-saturated thymine lesions in DNA: production by ultraviolet light or hydrogen peroxide. *Nucleic Acids Research*.

[6] Cathcart R, Schwiers E, Saul RL, Ames BN (1984). Thymine glycol and thymidine glycol in human and rat urine: a possible assay for oxidative DNA damage. *Proceedings of the National Academy of Sciences of the United States of America*.

[7] Harman D (1956). Aging: a theory based on free radical and radiation chemistry. *Journal of gerontology*.

[8] D’Autréaux B, Toledano MB (2007). ROS as signalling molecules: mechanisms that generate specificity in ROS homeostasis. *Nature Reviews Molecular Cell Biology*.

[9] Linnane AW, Kios M, Vitetta L (2007). Coenzyme Q10-Its role as a prooxidant in the formation of superoxide anion/hydrogen peroxide and the regulation of the metabolome. *Mitochondrion*.

[10] Nohl H, Kozlov AV, Staniek K, Gille L (2001). The multiple functions of coenzyme Q. *Bioorganic Chemistry*.

[11] Nohl H, Gille L, Staniek K (2005). Intracellular generation of reactive oxygen species by mitochondria. *Biochemical Pharmacology*.

[12] St-Pierre J, Buckingham JA, Roebuck SJ, Brand MD (2002). Topology of superoxide production from different sites in the mitochondrial electron transport chain. *The Journal of Biological Chemistry*.

[13] Jones DP (2008). Radical-free biology of oxidative stress. *American Journal of Physiology*.

[14] Klatt P, Lamas S (2000). Regulation of protein function by S-glutathiolation in response to oxidative and nitrosative stress. *European Journal of Biochemistry*.

[15] Fratelli M, Demol H, Puype M (2002). Identification by redox proteomics of glutathionylated proteins in oxidatively stressed human T lymphocytes. *Proceedings of the National Academy of Sciences of the United States of America*.

[16] Hisabori T, Hara S, Fujii T, Yamazaki D, Hosoya-Matsuda N, Motohashi K (2005). Thioredoxin affinity chromatography: a useful method for further understanding the thioredoxin network. *Journal of Experimental Botany*.

[17] Martínez-Ruiz A, Lamas S (2007). Proteomic identification of 5-nitrosylated proteins in endothelial cells. *Methods in Molecular Biology*.

[18] Martínez-Ruiz A, Lamas S (2007). Signalling by NO-induced protein S-nitrosylation and S-glutathionylation: convergences and divergences. *Cardiovascular Research*.

[19] Stadtman ER, Moskovitz J, Levine RL (2003). Oxidation of methionine residues of proteins: biological consequences. *Antioxidants and Redox Signaling*.

[20] Forman HJ, Fukuto JM, Torres M (2004). Redox signaling: thiol chemistry defines which reactive oxygen and nitrogen species can act as second messengers. *American Journal of Physiology*.

[21] Hess DT, Matsumoto A, Kim SO, Marshall HE, Stamler JS (2005). Protein S-nitrosylation: purview and parameters. *Nature Reviews Molecular Cell Biology*.

[22] Shechter Y, Burstein Y, Patchornik A (1975). Selective oxidation of methionine residues in proteins. *Biochemistry*.

[23] Veal EA, Day AM, Morgan BA (2007). Hydrogen peroxide sensing and signaling. *Molecular Cell*.

[24] Miki H, Funato Y (2012). Regulation of intracellular signalling through cysteine oxidation by reactive oxygen species. *Journal of Biochemistry*.

[25] Lambeth JD (2004). NOX enzymes and the biology of reactive oxygen. *Nature Reviews Immunology*.

[26] Linnane AW, Kios M, Vitetta L (2007). Healthy aging: regulation of the metabolome by cellular redox modulation and prooxidant signaling systems: the essential roles of superoxide anion and hydrogen peroxide. *Biogerontology*.

[27] Hansen JM, Watson WH, Jones DP (2004). Compartmentation of Nrf-2 redox control: regulation of cytoplasmic activation by glutathione and DNA binding by thioredoxin-1. *Toxicological Sciences*.

[28] Go YM, Jones DP (2008). Redox compartmentalization in eukaryotic cells. *Biochimica et Biophysica Acta*.

[29] Jones DP, Go YM (2010). Redox compartmentalization and cellular stress. *Diabetes, Obesity and Metabolism*.

[30] Taylor ER, Hurrell F, Shannon RJ, Lin TK, Hirst J, Murphy MP (2003). Reversible glutathionylation of complex I increases mitochondrial superoxide formation. *The Journal of Biological Chemistry*.

[31] Trotter EW, Grant CM (2003). Non-reciprocal regulation of the redox state of the glutathione-glutaredoxin and thioredoxin systems. *EMBO Reports*.

[32] Jones DP, Go YM, Anderson CL, Ziegler TR, Kinkade JM, Kirlin WG (2004). Cysteine/cystine couple is a newly recognized node in the circuitry for biologic redox signaling and control. *The FASEB Journal*.

[33] Hansen JM, Zhang H, Jones DP (2006). Differential oxidation of thioredoxin-1, thioredoxin-2, and glutathione by metal ions. *Free Radical Biology and Medicine*.

[34] Kemp M, Go YM, Jones DP (2008). Nonequilibrium thermodynamics of thiol/disulfide redox systems: a perspective on redox systems biology. *Free Radical Biology and Medicine*.

[35] Dwivedi G, Kemp ML (2012). Systemic redox regulation of cellular information processing. *Antioxidants and Redox Signaling*.

[36] Rhee SG, Bae YS, Lee SR, Kwon J (2000). Hydrogen peroxide: a key messenger that modulates protein phosphorylation through cysteine oxidation. *Science's STKE*.

[37] Meng TC, Fukada T, Tonks NK (2002). Reversible oxidation and inactivation of protein tyrosine phosphatases in vivo. *Molecular Cell*.

[38] Mahadev K, Zilbering A, Zhu L, Goldstein BJ (2001). Insulin-stimulated hydrogen peroxide reversibly inhibits protein-tyrosine phosphatase 1B in v ivo and enhances the early insulin action cascade. *The Journal of Biological Chemistry*.

[39] Kamata H, Honda SI, Maeda S, Chang L, Hirata H, Karin M (2005). Reactive oxygen species promote TNF*α*-induced death and sustained JNK activation by inhibiting MAP kinase phosphatases. *Cell*.

[40] Leslie NR, Bennett D, Lindsay YE, Stewart H, Gray A, Downes CP (2003). Redox regulation of PI 3-kinase signalling via inactivation of PTEN. *The EMBO Journal*.

[41] Laurent TC, Moore EC, Reichard P (1964). Enzymatic synthesis of deoxyribonucleotides. *The Journal of Biological Chemistry*.

[42] Moore EC, Reichard P, Thelander L (1964). Enzymatic synthesis of deoxyribonucleotides V. Purification and properties of thioredoxin reductase from Escherichia coli B. *The Journal of Biological Chemistry*.

[43] Pan JL, Bardwell JCA (2006). The origami of thioredoxin-like folds. *Protein Science*.

[44] Lillig CH, Holmgren A (2007). Thioredoxin and related molecules—from biology to health and disease. *Antioxidants and Redox Signaling*.

[45] Nakamura H, Nakamura K, Yodoi J (1997). Redox regulation of cellular activation. *Annual Review of Immunology*.

[46] Pekkari K, Holmgren A (2004). Truncated thioredoxin: physiological functions and mechanism. *Antioxidants and Redox Signaling*.

[47] Powis G, Montfort WR (2001). Properties and biological activities of thioredoxins. *Annual Review of Pharmacology and Toxicology*.

[48] Abate C, Patel L, Rauscher FJ, Curran T (1990). Redox regulation of Fos and Jun DNA-binding activity in vitro. *Science*.

[49] Okamoto T, Ogiwara H, Hayashi T, Mitsui A, Kawabe T, Yodoi J (1992). Human thioredoxin/adult T cell leukemia-derived factor activates the enhancer binding protein of human immunodeficiency virus type 1 by thiol redox control mechanism. *International Immunology*.

[50] Hayashi T, Ueno Y, Okamoto T (1993). Oxidoreductive regulation of nuclear factor *κ* B. Involvement of a cellular reducing catalyst thioredoxin. *The Journal of Biological Chemistry*.

[51] Kim SH, Oh J, Choi JY, Jang JY, Kang MW, Lee CE (2008). Identification of human thioredoxin as a novel IFN-gamma-induced factor: mechanism of induction and its role in cytokine production. *BMC Immunology*.

[52] Miranda-Vizuete A, González JCF, Gahmon G, Burghoorn J, Navas P, Swoboda P (2006). Lifespan decrease in a *Caenorhabditis elegans* mutant lacking TRX-1, a thioredoxin expressed in ASJ sensory neurons. *FEBS Letters*.

[53] Fierro-González JC, González-Barrios M, Miranda-Vizuete A, Swoboda P (2011). The thioredoxin TRX-1 regulates adult lifespan extension induced by dietary restriction in *Caenorhabditis elegans*. *Biochemical and Biophysical Research Communications*.

[54] Lim PIK, Liu J, Go MI, Boelsterli UA (2008). The mitochondrial superoxide/thioredoxin-2/ask1 signaling pathway is critically involved in troglitazone-induced cell injury to human hepatocytes. *Toxicological Sciences*.

[55] Schlotterer A, Hamann A, Kukudov G (2010). Apurinic/apyrimidinic endonuclease 1, p53, and thioredoxin are linked in control of aging in C. elegans. *Aging Cell*.

[56] Fierro-González JC, Cornils A, Alcedo J, Miranda-Vizuete A, Swoboda P (2011). The thioredoxin TRX-1 modulates the function of the insulin-like neuropeptide DAF-28 during dauer formation in *Caenorhabditis elegans*. *PLoS ONE*.

[57] Funato Y, Miki H (2007). Nucleoredoxin, a novel thioredoxin family member involved in cell growth and differentiation. *Antioxidants and Redox Signaling*.

[58] Hirota K, Matsui M, Murata M (2000). Nucleoredoxin, glutaredoxin, and thioredoxin differentially regulate NF-*κ*B, AP-1, and CREB activation in HEK293 cells. *Biochemical and Biophysical Research Communications*.

[59] Hayashi T, Funato Y, Terabayashi T (2010). Nucleoredoxin negatively regulates toll-like receptor 4 signaling via recruitment of flightless-I to myeloid differentiation primary response gene (88). *The Journal of Biological Chemistry*.

[60] Funato Y, Terabayashi T, Sakamoto R (2010). Nucleoredoxin sustains Wnt/*β*-catenin signaling by retaining a pool of inactive dishevelled protein. *Current Biology*.

[61] Kunchithapautham K, Padmavathi B, Narayanan RB, Kaliraj P, Scott AL (2003). Thioredoxin from Brugia malayi: defining a 16-kilodalton class of thioredoxins from nematodes. *Infection and Immunity*.

[62] Hatahet F, Ruddock LW (2009). Protein disulfide isomerase: a critical evaluation of its function in disulfide bond formation. *Antioxidants and Redox Signaling*.

[63] Pompella A, Visvikis A, Paolicchi A, De Tata V, Casini AF (2003). The changing faces of glutathione, a cellular protagonist. *Biochemical Pharmacology*.

[64] Schafer FQ, Buettner GR (2001). Redox environment of the cell as viewed through the redox state of the glutathione disulfide/glutathione couple. *Free Radical Biology and Medicine*.

[65] Sheehan D, Meade G, Foley VM, Dowd CA (2001). Structure, function and evolution of glutathione transferases: implications for classification of non-mammalian members of an ancient enzyme superfamily. *Biochemical Journal*.

[66] Paulsen CE, Carroll KS (2010). Orchestrating redox signaling networks through regulatory cysteine switches. *ACS Chemical Biology*.

[67] Hearne JL, Colman RF (2005). Delineation of xenobiotic substrate sites in rat glutathione S-transferase M1-1. *Protein Science*.

[68] Chaudhari A, Anderson MW, Eling TE (1978). Conjugation of 15-keto-prostaglandins by glutathione S-transferases. *Biochimica et Biophysica Acta*.

[69] Christ-Hazelhof E, Nugteren DH, Van Dorp DA (1976). Conversions of prostaglandin endoperoxides by glutathione S transferases and serum albumins. *Biochimica et Biophysica Acta*.

[70] Hwang C, Sinskey AJ, Lodish HF (1992). Oxidized redox state of glutathione in the endoplasmic reticulum. *Science*.

[71] Baryshev M, Sargsyan E, Mkrtchian S (2006). ERp29 is an essential endoplasmic reticulum factor regulating secretion of thyroglobulin. *Biochemical and Biophysical Research Communications*.

[72] Sargsyan E, Baryshev M, Szekely L, Sharipo A, Mkrtchian S (2002). Identification of ERp29, an endoplasmic reticulum lumenal protein, as a new member of the thyroglobulin folding complex. *The Journal of Biological Chemistry*.

[73] Desilva MG, Lu J, Donadel G (1996). Characterization and chromosomal localization of a new protein disulfide isomerase, PDIp, highly expressed in human pancreas. *DNA and Cell Biology*.

[74] Desilva MG (1997). Molecular characterization of a pancreas-specific protein disulfide isomerase, PDIp. *DNA and Cell Biology*.

[75] Peaper DR, Cresswell P (2008). Regulation of MHC class I assembly and peptide binding. *Annual Review of Cell and Developmental Biology*.

[76] Mezghrani A, Fassio A, Benham A, Simmen T, Braakman I, Sitia R (2001). Manipulation of oxidative protein folding and PDI redox state in mammalian cells. *The EMBO Journal*.

[77] Zechmann B, Mauch F, Sticher L, Müller M (2008). Subcellular immunocytochemical analysis detects the highest concentrations of glutathione in mitochondria and not in plastids. *Journal of Experimental Botany*.

[78] Foyer CH, Noctor G (2005). Redox homeostasis and antioxidant signaling: a metabolic interface between stress perception and physiological responses. *The Plant Cell*.

[79] Mou Z, Fan W, Dong X (2003). Inducers of plant systemic acquired resistance regulate NPR1 function through redox changes. *Cell*.

[80] Gomez LD, Noctor G, Knight MR, Foyer CH (2004). Regulation of calcium signalling and gene expression by glutathione. *Journal of Experimental Botany*.

[81] Mustacich D, Powis G (2000). Thioredoxin reductase. *Biochemical Journal*.

[82] Kanzok SM, Fechner A, Bauer H (2001). Substitution of the thioredoxin system for glutathione reductase in *Drosophila melanogaster*. *Science*.

[83] Pedrajas JR, Kosmidou E, Miranda-Vizuete A, Gustafsson JA, Wright APH, Spyrou G (1999). Identification and functional characterization of a novel mitochondrial thioredoxin system in *Saccharomyces cerevisiae*. *The Journal of Biological Chemistry*.

[84] Stenvall J, Fierro-González JC, Swoboda P (2011). Selenoprotein TRXR-1 and GSR-1 are essential for removal of old cuticle during molting in *Caenorhabditis elegans*. *Proceedings of the National Academy of Sciences of the United States of America*.

[85] Bunik VI (2003). 2-Oxo acid dehydrogenase complexes in redox regulation: role of the lipoate residues and thioredoxin. *European Journal of Biochemistry*.

[86] Tretter L, Adam-Vizi V (2004). Generation of reactive oxygen species in the reaction catalyzed by *α*-ketoglutarate dehydrogenase. *Journal of Neuroscience*.

[87] Starkov AA, Fiskum G, Chinopoulos C (2004). Mitochondrial *α*-ketoglutarate dehydrogenase complex generates reactive oxygen species. *Journal of Neuroscience*.

[88] Meyer AJ, Brach T, Marty L (2007). Redox-sensitive GFP in Arabidopsis thaliana is a quantitative biosensor for the redox potential of the cellular glutathione redox buffer. *Plant Journal*.

[89] Fernandes AP, Holmgren A (2004). Glutaredoxins: glutathione-dependent redox enzymes with functions far beyond a simple thioredoxin backup system. *Antioxidants and Redox Signaling*.

[90] Haunhorst P, Berndt C, Eitner S, Godoy JR, Lillig CH (2010). Characterization of the human monothiol glutaredoxin 3 (PICOT) as iron-sulfur protein. *Biochemical and Biophysical Research Communications*.

[91] Cheng NH (2009). Picot, a novel monothiol glutaredoxin, plays a role in postembryonic growth and cardiac function in mice under nutritional perturbation. *Journal of Federation of American Societies for Experimental Biology*.

[92] Shelton MD, Chock PB, Mieyal JJ (2005). Glutaredoxin: role in reversible protein S-glutathionylation and regulation of redox signal transduction and protein translocation. *Antioxidants and Redox Signaling*.

[93] Sykes MC, Mowbray AL, Jo H (2007). Reversible glutathiolation of caspase-3 by glutaredoxin as a novel redox signaling mechanism in tumor necrosis factor-*α*-induced cell death. *Circulation Research*.

[94] Adachi T, Pimentel DR, Heibeck T (2004). S-glutathiolation of Ras mediates redox-sensitive signaling by angiotensin II in vascular smooth muscle cells. *The Journal of Biological Chemistry*.

[95] Morgan KL, Estevez AO, Mueller CL (2010). The glutaredoxin GLRX-21 functions to prevent selenium-induced oxidative stress in *Caenorhabditis elegans*. *Toxicological Sciences*.

[96] Wilkinson B, Gilbert HF (2004). Protein disulfide isomerase. *Biochimica et Biophysica Acta*.

[97] Bass R, Ruddock LW, Klappa P, Freedman RB (2004). A major fraction of endoplasmic reticulum-located glutathione is present as mixed disulfides with protein. *The Journal of Biological Chemistry*.

[98] Karala AR, Psarrakos P, Ruddock LW, Klappa P (2007). Protein disulfide isomerases from C. elegans are equally efficient at thiol-disulfide exchange in simple peptide-based systems but show differences in reactivity towards protein substrates. *Antioxidants and Redox Signaling*.

[99] Winter AD, McCormack G, Page AP (2007). Protein disulfide isomerase activity is essential for viability and extracellular matrix formation in the nematode *Caenorhabditis elegans*. *Developmental Biology*.

[100] Alanen HI, Salo KEH, Pirneskoski A, Ruddock LW (2006). pH dependence of the peptide thiol-disulfide oxidase activity of six members of the human protein bisulfide isomerase family. *Antioxidants and Redox Signaling*.

[101] Winter AD, Page AP (2000). Prolyl 4-hydroxylase is an essential procollagen-modifying enzyme required for exoskeleton formation and the maintenance of body shape in the nematode *Caenorhabditis elegans*. *Molecular and Cellular Biology*.

[102] Keskiaho K, Kukkola L, Page AP (2008). Characterization of a novel *Caenorhabditis elegans* prolyl 4-hydroxylase with a unique substrate specificity and restricted expression in the pharynx and excretory duct. *The Journal of Biological Chemistry*.

[103] Veijola J, Annunen P, Koivunen P, Page AP, Pihlajaniemi T, Kivirikko KI (1996). Baculovirus expression of two protein disulphide isomerase isoforms from *Caenorhabditis elegans* and characterization of prolyl 4-hydroxylases containing one of these polypeptides as their *β* subunit. *Biochemical Journal*.

[104] Ko FCF, Chow KL (2002). A novel thioredoxin-like protein encoded by the C. elegans dpy-11 gene is required for body and sensory organ morphogenesis. *Development*.

[105] Higuchi T, Watanabe Y, Waga I (2004). Protein disulfide isomerase suppresses the transcriptional activity of NF-*κ*B. *Biochemical and Biophysical Research Communications*.

[106] Babior BM, Lambeth JD, Nauseef W (2002). The neutrophil NADPH oxidase. *Archives of Biochemistry and Biophysics*.

[107] Moreno JC, Bikker H, Kempers MJE (2002). Inactivating mutations in the gene for thyroid oxidase 2 (THOX2) and congenital hypothyroidism. *The New England Journal of Medicine*.

[108] Edens WA, Sharling L, Cheng G (2001). Tyrosine cross-linking of extracellular matrix is catalyzed by Duox, a multidomain oxidase/peroxidase with homology to the phagocyte oxidase subunit gp91phox. *Journal of Cell Biology*.

[109] Meitzler JL, Brandman R, Ortiz De Montellano PR (2010). Perturbed heme binding is responsible for the blistering phenotype associated with mutations in the *Caenorhabditis elegans* dual oxidase 1 (DUOX1) peroxidase domain. *The Journal of Biological Chemistry*.

[110] Buettner GR, Ng CF, Wang M, Rodgers VGJ, Schafer FQ (2006). A New paradigm: manganese superoxide dismutase influences the production of H_2_O_2_ in cells and thereby their biological state. *Free Radical Biology and Medicine*.

[111] Longo VD, Gralla EB, Valentine JS (1996). Superoxide dismutase activity is essential for stationary phase survival in *Saccharomyces cerevisiae*: mitochondrial production of toxic oxygen species in vivo. *The Journal of Biological Chemistry*.

[112] Longo VD, Liou LL, Valentine JS, Gralla EB (1999). Mitochondrial superoxide decreases yeast survival in stationary phase. *Archives of Biochemistry and Biophysics*.

[113] Wawryn J, Krzepiłko A, Myszka A, Biliński T (1999). Deficiency in superoxide dismutases shortens life span of yeast cells. *Acta Biochimica Polonica*.

[114] Unlu ES, Koc A (2007). Effects of deleting mitochondrial antioxidant genes on life span. *Annals of the New York Academy of Sciences*.

[115] Phillips JP, Campbell SD, Michaud D, Charbonneau M, Hilliker AJ (1989). Null mutation of copper/zinc superoxide dismutase in Drosophila confers hypersensitivity to paraquat and reduced longevity. *Proceedings of the National Academy of Sciences of the United States of America*.

[116] Elchuri S, Oberley TD, Qi W (2005). CuZnSOD deficiency leads to persistent and widespread oxidative damage and hepatocarcinogenesis later in life. *Oncogene*.

[117] Doonan R, McElwee JJ, Matthijssens F (2008). Against the oxidative damage theory of aging: superoxide dismutases protect against oxidative stress but have little or no effect on life span in *Caenorhabditis elegans*. *Genes and Development*.

[118] Seto NOL, Hayashi S, Tener GM (1990). Overexpression of Cu-Zn superoxide dismutase in Drosophila does not affect life-span. *Proceedings of the National Academy of Sciences of the United States of America*.

[119] Staveley BE, Phillips JP, Hilliker AJ (1990). Phenotypic consequences of copper-zinc superoxide dismutase overexpression in *Drosophila melanogaster*. *Genome*.

[120] Van Raamsdonk JM, Hekimi S (2009). Deletion of the mitochondrial superoxide dismutase sod-2 extends lifespan in *Caenorhabditis elegans*. *PLoS Genetics*.

[121] Honda Y, Honda S (1999). The daf-2 gene network for longevity regulates oxidative stress resistance and Mn-superoxide dismutase gene expression in *Caenorhabditis elegans*. *The FASEB Journal*.

[122] Liochev SI, Fridovich I (2010). Mechanism of the peroxidase activity of Cu, Zn superoxide dismutase. *Free Radical Biology and Medicine*.

[123] Holley AK, Bakthavatchalu V, Velez-Roman JM, St Clair DK (2011). Manganese superoxide dismutase: guardian of the powerhouse. *International Journal of Molecular Sciences*.

[124] Fujii M, Ishii N, Joguchi A, Yasuda K, Ayusawa D (1998). A novel superoxide dismutase gene encoding membrane-bound and extracellular isoforms by alternative splicing in *Caenorhabditis elegans*. *DNA Research*.

[125] Lubos E, Loscalzo J, Handy DE (2011). Glutathione peroxidase-1 in health and disease: from molecular mechanisms to therapeutic opportunities. *Antioxidants and Redox Signaling*.

[126] Vanfleteren JR (1993). Oxidative stress and ageing in *Caenorhabditis elegans*. *Biochemical Journal*.

[127] Maiorino M, Ursini F, Bosello V (2007). The thioredoxin specificity of Drosophila GPx: a paradigm for a peroxiredoxin-like mechanism of many glutathione peroxidases. *Journal of Molecular Biology*.

[128] Faltin Z, Holland D, Velcheva M (2010). Glutathione peroxidase regulation of reactive oxygen species level is crucial for in vitro plant differentiation. *Plant and Cell Physiology*.

[129] Lubos E, Mahoney CE, Leopold JA, Zhang YY, Loscalzo J, Handy DE (2010). Glutathione peroxidase-1 modulates lipopolysaccharide-induced adhesion molecule expression in endothelial cells by altering CD14 expression. *The FASEB Journal*.

[130] Lubos E, Kelly NJ, Oldebeken SR (2011). Glutathione peroxidase-1 deficiency augments proinflammatory cytokine-induced redox signaling and human endothelial cell activation. *The Journal of Biological Chemistry*.

[131] Carter AB, Tephly LA, Venkataraman S (2004). High levels of catalase and glutathione peroxidase activity dampen H_2_O_2_ signaling in human alveolar macrophages. *American Journal of Respiratory Cell and Molecular Biology*.

[132] Benner J, Daniel H, Spanier B (2011). A glutathione peroxidase, intracellular peptidases and the TOR complexes regulate peptide transporter PEPT-1 in C. elegans. *PloS one*.

[133] Sue GR, Ho ZC, Kim K (2005). Peroxiredoxins: a historical overview and speculative preview of novel mechanisms and emerging concepts in cell signaling. *Free Radical Biology and Medicine*.

[134] Palande K, Roovers O, Gits J (2011). Peroxiredoxin-controlled G-CSF signalling at the endoplasmic reticulum-early endosome interface. *Journal of Cell Science*.

[135] Isermann K, Liebau E, Roeder T, Bruchhaus I (2004). A peroxiredoxin specifically expressed in two types of pharyngeal neurons is required for normal growth and egg production in *Caenorhabditis elegans*. *Journal of Molecular Biology*.

[136] Oláhová M, Taylor SR, Khazaipoul S (2008). A redox-sensitive peroxiredoxin that is important for longevity has tissue- and stress-specific roles in stress resistance. *Proceedings of the National Academy of Sciences of the United States of America*.

[137] Taub J, Lau JF, Ma C (1999). A cytosolic catalase is needed to extend adult lifespan in C. elegans daf-C and clk-1 mutants. *Nature*.

[138] Togo SH, Maebuchi M, Yokota S, Bun-ya M, Kawahara A, Kamiryo T (2000). Immunological detection of alkaline-diaminobenzidine-negative peroxisomes of the nematode *Caenorhabditis elegans*: purification and unique pH optima of peroxisomal catalase. *European Journal of Biochemistry*.

[139] Petriv OI, Rachubinski RA (2004). Lack of peroxisomal catalase causes a progeric phenotype in *Caenorhabditis elegans*. *The Journal of Biological Chemistry*.

[140] Murphy CT, McCarroll SA, Bargmann CI (2003). Genes that act downstream of DAF-16 to influence the lifespan of *Caenorhabditis elegans*. *Nature*.

